# Summary of Expert Opinion on the Management of Children With Chronic Kidney Disease and Growth Failure With Human Growth Hormone

**DOI:** 10.3389/fendo.2020.00587

**Published:** 2020-09-02

**Authors:** Marco Cappa, Mohamad Maghnie, Vincenza Carbone, Laura Chioma, Carmela Errichiello, Claudia Giavoli, Mario Giordano, Laura Guazzarotti, Antonella Klain, Giovanni Montini, Luisa Murer, Maria Parpagnoli, Carmine Pecoraro, Sabino Pesce, Enrico Verrina

**Affiliations:** ^1^Unit of Endocrinology, Bambino Gesù Children's Hospital, IRCCS, Rome, Italy; ^2^Department of Pediatrics, IRCCS Istituto Giannina Gaslini, University of Genova, Genova, Italy; ^3^Department of Neuroscience, Rehabilitation, Ophtalmology, Genetics, Maternal and Child Health, University of Genova, Genova, Italy; ^4^Pediatric Nephrology and Dialysis Unit, Pediatric Hospital “Giovanni XXIII”, Bari, Italy; ^5^Nephrology Unit, Meyer Children's University Hospital, Florence, Italy; ^6^Endocrinology Unit, Fondazione IRCCS Ca' Granda Ospedale Maggiore Policlinico, Department of Clinical Sciences and Community Health, University of Milan, Milan, Italy; ^7^Pediatric Endocrinology and Auxology, Adolescence Unit - Department of Women's and Children's Health, Pediatric Department - Padua University Hospital, Padua, Italy; ^8^Pediatric Endocrinology Unit, Santobono-Pausilipon Hospital, Naples, Italy; ^9^Pediatric Nephrology, Dialysis, and Transplant Unit, Fondazione IRCCS Ca' Granda Ospedale Maggiore Policlinico, Milan, Italy; ^10^Giuliana and Bernardo Caprotti Chair of Pediatrics, Department of Clinical Sciences and Community Health, University of Milan, Milan, Italy; ^11^Pediatric Nephrology, Dialysis and Transplant Unit, Department of Woman's and Child's Health, Azienda Ospedaliera –University of Padova, Padua, Italy; ^12^Auxo-Endocrinology and Gynecology Meyer Children's University Hospital, Florence, Italy; ^13^Pediatric Nephrology and Dialysis Unit, Santobono-Pausilipon Hospital, Naples, Italy; ^14^Pediatric Endocrinology and Metabolic Diseases, Pediatric Hospital “Giovanni XXIII”, Bari, Italy; ^15^Unit of Dialysis, IRCCS Istituto Giannina Gaslini, Genoa, Italy

**Keywords:** growth, growth hormone, final height, chronic kidney disease, GFR – glomerular filtration rate, kidney transplant, hyperparathyroidism

## Abstract

**Background:** The management of children and adolescents with chronic kidney disease (CKD) and growth failure candidate for recombinant human growth hormone therapy (rhGH) is based on an appraisal of the literature established on a 2006 consensus statement and 2019 Clinical practice recommendations. The performance of these guidelines has never been tested.

**Aims:** The objective of this study was to establish the level of adherence to international guidelines based on the 2006 consensus and the 2019 criteria that lead to the initiation of growth hormone treatment by both pediatric endocrinologists and pediatric nephrologists.

**Methods:** A multidisciplinary team of pediatric endocrinologists and pediatric nephrologists, members of the Italian Society of Pediatric Endocrinology or of the Italian Society of Pediatric Nephrology, discussed and reviewed the main issues related to the management of pediatric patients with CKD who need treatment with rhGH. Experts developed 11 questions focusing on risk assessment and decision makings in October 2019 and a survey was sent to forty pediatric endocrinologists (*n* = 20) and nephrologists (*n* = 20) covering the whole national territory. The results were then analyzed and discussed in light of current clinical practice guidelines and recent recommendations.

**Results:** Responses were received from 32 of the 40 invited specialists, 17 of whom were pediatric endocrinologists (42.5%) and 15 pediatric nephrologists (37.5%). Although all the centers that participated in the survey agreed to follow the clinical and biochemical diagnostic work-up and the criteria for the treatment of patients with CKD, among the Italian centers there was a wide variety of decision-making processes.

**Conclusions:** Despite current guidelines for the management of children with CKD and growth failure, its use varies widely between centers and rhGH is prescribed in a relatively small number of patients and rarely after kidney transplantation. Several raised issues are not taken into account by international guidelines and a multidisciplinary approach with mutual collaboration between specialists will improve patient care based on their unmet needs.

## Introduction

Chronic Kidney Disease (CKD) in pediatric and adult patients is characterized by a progressive decline in kidney function, classified according to the Kidney Disease: Improving Global Outcomes (KDIGO) in a system based primarily on glomerular filtration rate (eGFR, ml/min/1.73 m^2^) and on proteinuria (1). The classification consists of 5 stages based on a decreasing of eGFR: G1 normal or high (≥ 90); G2 mildly decreased (60–89); G3a mildly to moderately decreased (45–59); G3b moderately to severely decreased (30–44); G4 severely decreased (15–29) and G5 kidney failure (≤ 15, or dialysis) (1). According to these Guidelines, CKD should be established based on kidney function and damage expressed as eGFR, irrespective of diagnosis. Therefore, a patient has CKD if either of these criteria are fulfilled:

1. Kidney damage for 3 months defined by structural or functional abnormalities of the kidney, with or without decreased eGFR, manifested by 1 or more of the following features:
  - Abnormalities in the composition of the blood or urine (CKD guidelines emphasize persistent proteinuria as a particularly important marker of kidney damage)
  - Abnormalities in imaging investigations or on kidney biopsy
2. eGFR of <60 mL/min/1.73 m^2^ for 3 months, with or without the other signs of kidney damage (1).

The rationale for including individuals with normal eGFR is that substantial kidney damage often occurs before this pivotal component of kidney function declines, and that these individuals are at increased risk for adverse outcomes of CKD (1).

Growth failure in CKD patients has multifactorial origins including intrauterine growth restriction, malnutrition, mineral and bone disease secondary to CKD, metabolic acidosis, electrolyte disturbances, and changes of hypothalamic-pituitary-somatotropic and -gonadal axes (2). The evaluation of growth retardation and protein-energy malnutrition, common clinical features in children with CKD, plays an essential role within routine pediatric nephrology care (3). About 40% of children with End Stage Renal Disease (ESRD) have an adult height below the third percentile compared to healthy young-adult subjects of similar age and gender (4, 5). Furthermore, short stature correlates with a worsening in quality of life, self-esteem and social inclusion, while height gain and recombinant human GH (rhGH) use are associated with improvement of physical and social functioning (6–8).

Factors that contribute to growth failure in children with CKD are manifold and well-described in a recent consensus (2) (Table 1). In particular, key factors that mainly influence growth of children and adolescents with CKD are represented by nutrition, the GH-IGF-I axis, and steroids at puberty (9, 10). Nutritional deficiency is a major cause of growth failure in infancy and early childhood (11). According to Behnisch et al. other factors such as parental height, pubertal status, body mass index (BMI) and the country of origin may affect growth in children with CKD, while anemia, metabolic acidosis and chronic Kidney disease mineral bone disorder (CKD-MBD) appear to have less relevant impact (12). In addition, GH and IGF-I are determinants of longitudinal bone growth, skeletal maturation, muscle and fat metabolism, and acquisition of bone mass during the pubertal period, whereas in adults they are important in the maintenance of bone mass and metabolism processes (13–15).

**Table 1:** Factors that contribute to growth failure in children with CKD.

**Genetic factors, (gender, target height)**	**Malnutrition, anemia**	**Mineral bone disorder**
Birth-related factors (prematurity, small for gestational age)	Metabolic acidosis	Age at onset of CKD
Non-renal comorbidities, infections	Hormonal disturbances (GH-IGF-I axis, PTH)	CKD stage, residual renal function in patients on dialysis

A variable degree of GH insensitivity has been reported in advanced CKD conditions (16, 17). Such insensitivity could be partially compensated by the administration of supraphysiological doses of rhGH, which can stimulate the synthesis of IGF1 and promote longitudinal growth (18, 19). Nissel et al. state that growth response to GH is significantly associated with the initial degree of stunting, time spent on conservative therapy/dialysis, delayed bone age, pubertal delay, gender and age at the start of rhGH treatment, and duration of rhGH therapy (20).

In children with CKD and growth failure, the main rhGH treatment objectives could be summarized by correction of metabolic abnormalities, improving the growth rate [cm/year; Standard Deviation Score (SDS)], normalizing the growth pattern, and achieving the normal adult height (19). Successful therapy is based on early diagnosis and intervention, as well as on optimal adherence to therapy (21). Patients adhering to rhGH treatment demonstrated a higher rate of growth than non-adherent patients, highlighting how non-adherence should be considered and evaluated in the context of poor and/or no response to rhGH therapy in patients with CKD (22).

## Materials and Methods

The main criterion for conducting the survey was to involve experts among the Italian Society of Pediatric Nephrology (Società Italiana di Nefrologia Pediatrica- SINePe; small society) and the Society of Pediatric Endocrinology (Società Italiana di Endocrinologia e Diabetologia Pediatrica-SIEDP; larger society) who collaborate together as a multidisciplinary team (MDT) in the care of children and adolescents with CKD (dialysis, Kidney transplantation) and growth failure, compared to the few others who work in a non-multidisciplinary approach. Among the National centers, 7 Pediatric nephrologists from Padua, Milan, Genoa, Florence, Rome, Naples and Bari joined the Steering Committee, while the remaining two have answered to the questionnaires (Italy have 5 national centers for kidney transplantation); the number of pediatric endocrinologists was then adapted. Thus, the participating centers represent the majority of Italian Regions from the North to the South with comparable healthcare systems facing similar challenges regarding the management of patients with CKD and growth failure.

This study is pinpointed on an expert opinion approach in the evaluation of CKD children potentially candidates for treatment with rhGH based on current knowledge.

A steering committee of 11 experts met in nominal group format to prioritize questions to be addressed and identify core bibliographic materials and criteria for survey panelists. The members of the scientific committee have been identified on a multidisciplinary approach team basis to foster collaboration between pediatric endocrinologists and pediatric nephrologists.

In a first step a targeted literature search of PubMed (search term: CKD and growth failure) was used to generate core bibliographic material and proposed to the steering committee together with a brief context analysis on the use of rhGH in children (national rules and distribution, prescribing centers) and with particular attention to the management of pediatric patients with CKD and growth failure who may need rhGH treatment. The list of references is available in supplemental materials.

In October 2019, a steering committee first round was conducted with a discussion in a face-to-face focus group based on reviewing the best available evidence and recommendations, and the combination of expert experience, following the principle of “evidence-based, consensus-based and experience-based.” The committee also evaluated topics related to the disparity between guidelines and clinical practice and compared different views on the management of children with CKD and growth failure between nephrologists and endocrinologists; they also discussed the role of restrictions related to the Italian rules on the prescription of rhGH.

During this meeting the steering committee developed 11 questions to assess the physicians' orientation toward the use of rhGH in children with CKD and growth failure, to evaluate different clinical approach between the specialists, to explore risk assessment and decision analysis, and to evaluate adherence to guidelines in real life. The 11 questions were then administered in a web-based survey to members of the scientific committee and to an expanded panel of endocrinologists and nephrologists chosen from among the members of the 2 scientific societies uniformly distributed across the country, even when both hospital units (endocrinology and nephrology units) were not available in the same Center. The questionnaire was administered using Survey Monkey (Survey Monkey, Palo Alto, CA, USA), and the survey link was disseminated to the participants via a dedicated email indicating to avoid the use of personal information and that and that the information that could allow identification will not be used for the data analysis.

Participants were given a response deadline of 5 weeks after receiving the survey. The survey was made available online from 29 October 2019 to 04 December 2019. The survey was completed by 32 of the 40 invited specialists, 17 of whom were pediatric endocrinologists (42.5%) and 15 pediatric nephrologists (37.5%).

The data collected through the survey were processed to investigate the response rates of the individual subgroups interviewed; comparative analyzes were conducted between the different subgroups belonging to the sample, in order to highlight the results that emerged through the application of modeling filters. The description of the data also included proportions, frequencies, averages, and standard deviations when appropriate. The descriptive statistics were used to summarize the entire data and discussed in December 2019 in a second face-to-face meeting.

Three members of the scientific committee (MC, MM, EV) drew up a document that summarized the picture of the management of growth failure in children with CKD in Italy on the basis of the results of the questionnaires comparing it with the current international guidelines; the document was then approved by the members of the steering committee.

## Results

**Question 1**. When do you think it is necessary to start treatment with rhGH in children younger than 2 years old with CKD and growth failure, once other potentially treatable risk factors related to growth failure and/or reduced growth rate have been adequately addressed?

**Figure 1:**
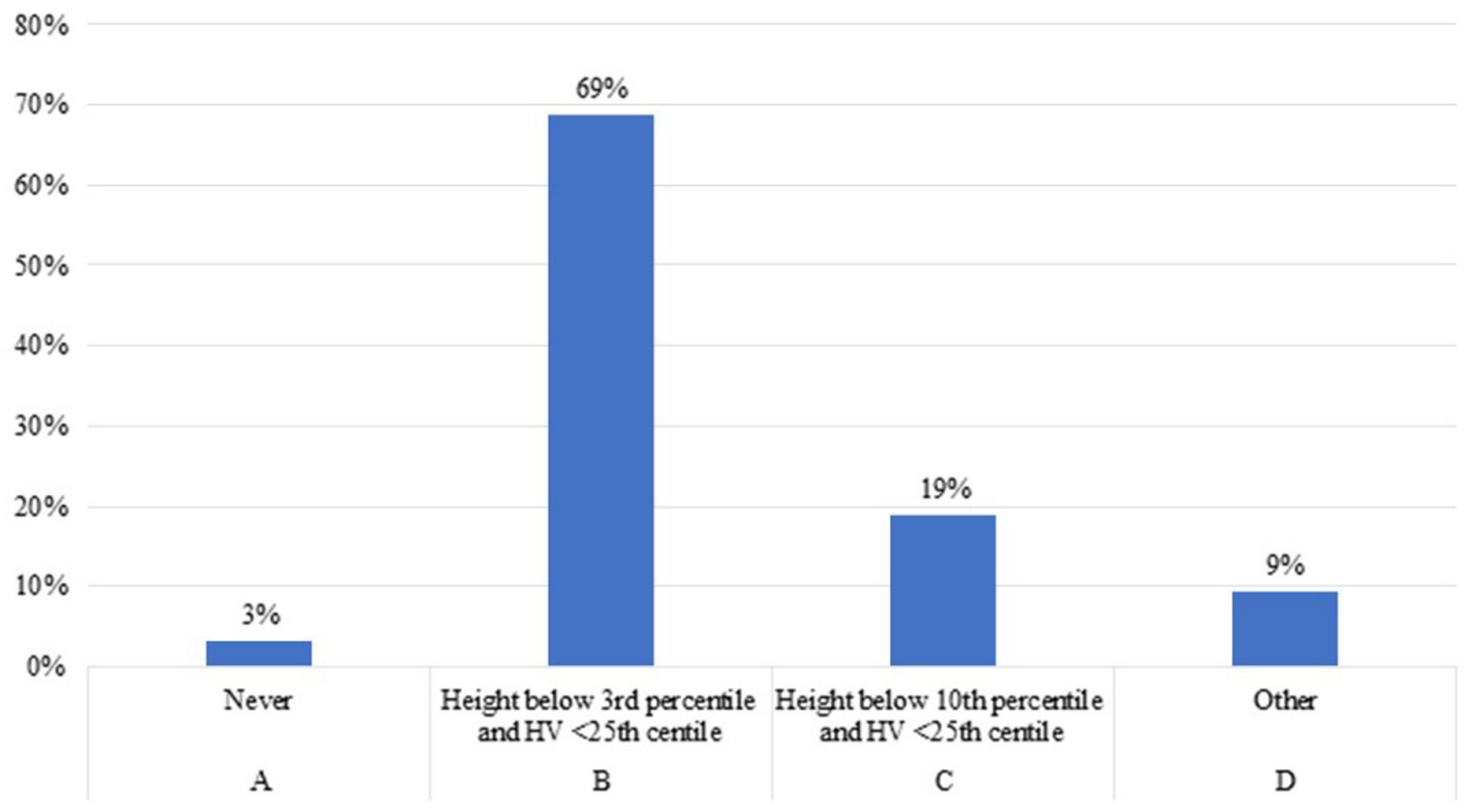
Height parameters used to start rhGH treatment in patient below 2 years old with CKD.

Eighty-eight percent of the experts agreed to start rhGH treatment in patients younger than 2 years with CKD and growth failure, once other potentially treatable risk factors related to growth failure and/or reduced growth rate have been adequately addressed (Figures 1B,C). Both expert specialists believed that it is important to start rhGH treatment when height is below the third percentile and height velocity (HV) is below the twenty- fifth percentile (Figure 1). Experts highlights that optimal nutritional management could be followed by catch-up growth, even in infants with severe CKD (23, 24). However, some of these patients have growth failure despite an adequate energy and protein supply, especially those with CKD stage 3–5 and on dialysis.

The experts opinion is in line with the consensus statement by Mahan et al. where is reported that, since children attain one-third of their adult height during the first 2 years of life, growth impairment during infancy has a greater impact on adult stature than later-onset diseases may have. Nevertheless, CKD affects growth throughout childhood and is associated with a delayed pubertal growth spurt and reduced pubertal height gain (25).

**Question 2**. When do you start rhGH treatment in children with CKD younger than 2 years, once other factors related to poor weight gain have been addressed?

**Figure 2:**
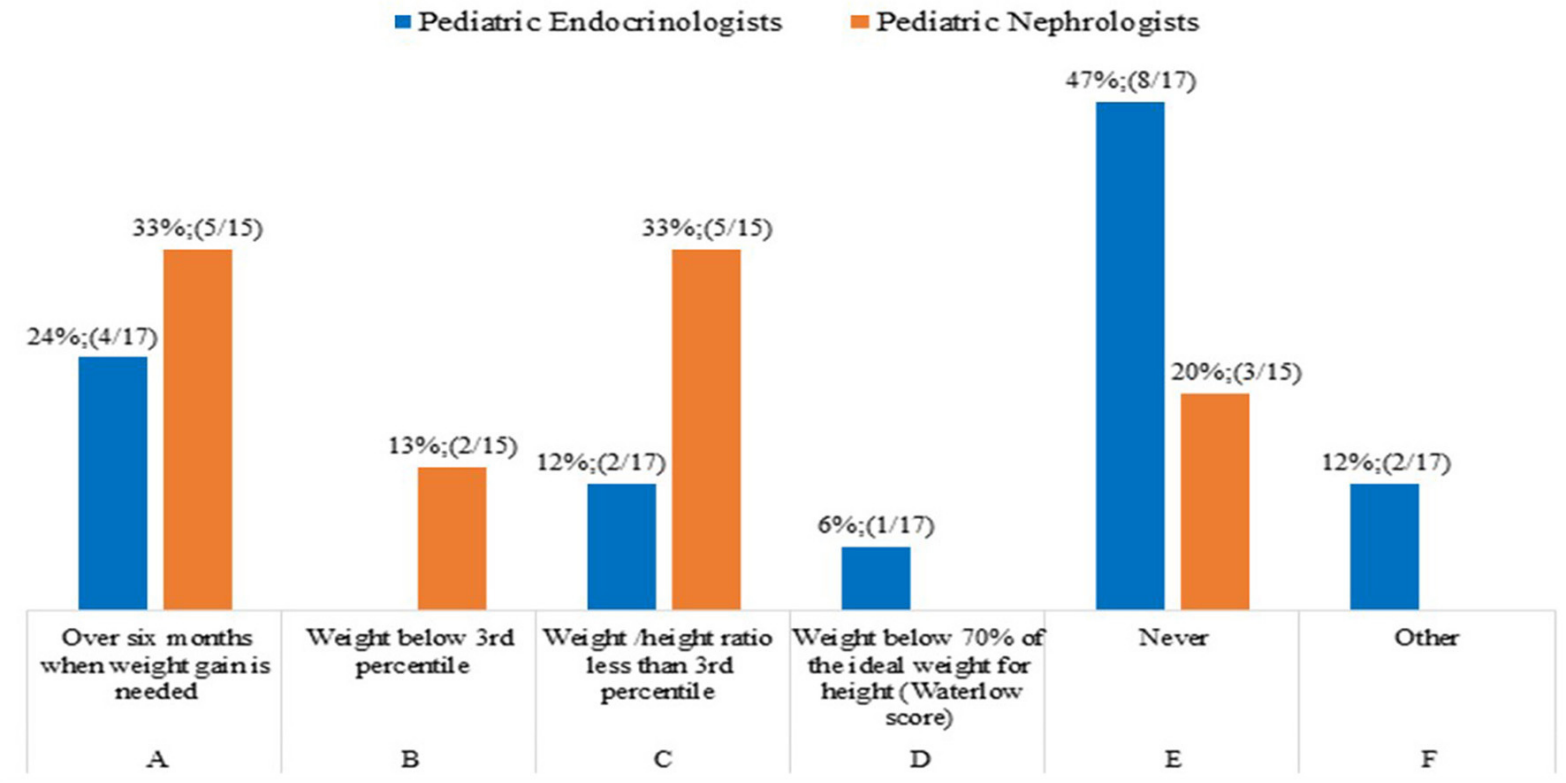
rhGH treatment in children (<2 years old) with CKD and weight gain.

The common clinical practice of 47% of pediatric Endocrinologists (8/17) was not to treat children with CKD and growth failure younger than 2 years, on the basis of weight status i.e., a reduced weight gain, since the decision whether to treat or not treat is primarily based on the height rather than weight parameter (Figure 2).

Recent studies suggested that rhGH treatment should be started early in infants with nephropathic cystinosis if growth failure is detected despite adequate nutrition and cysteamine treatment both during conservative treatment and during renal replacement therapy. It is also assumed that nutrition adequacy is essential to promote growth and development of children with CKD. However, it has been demonstrated that aggressive enteral feeding in infants with CKD stage 5 on peritoneal dialysis may result in excessive weight gain with rather limited effects on longitudinal growth (26, 27).

**Question 3**. When do you think it is necessary to start rhGH treatment in children with growth failure and CKD older than 2 years?

**Figure 3:**
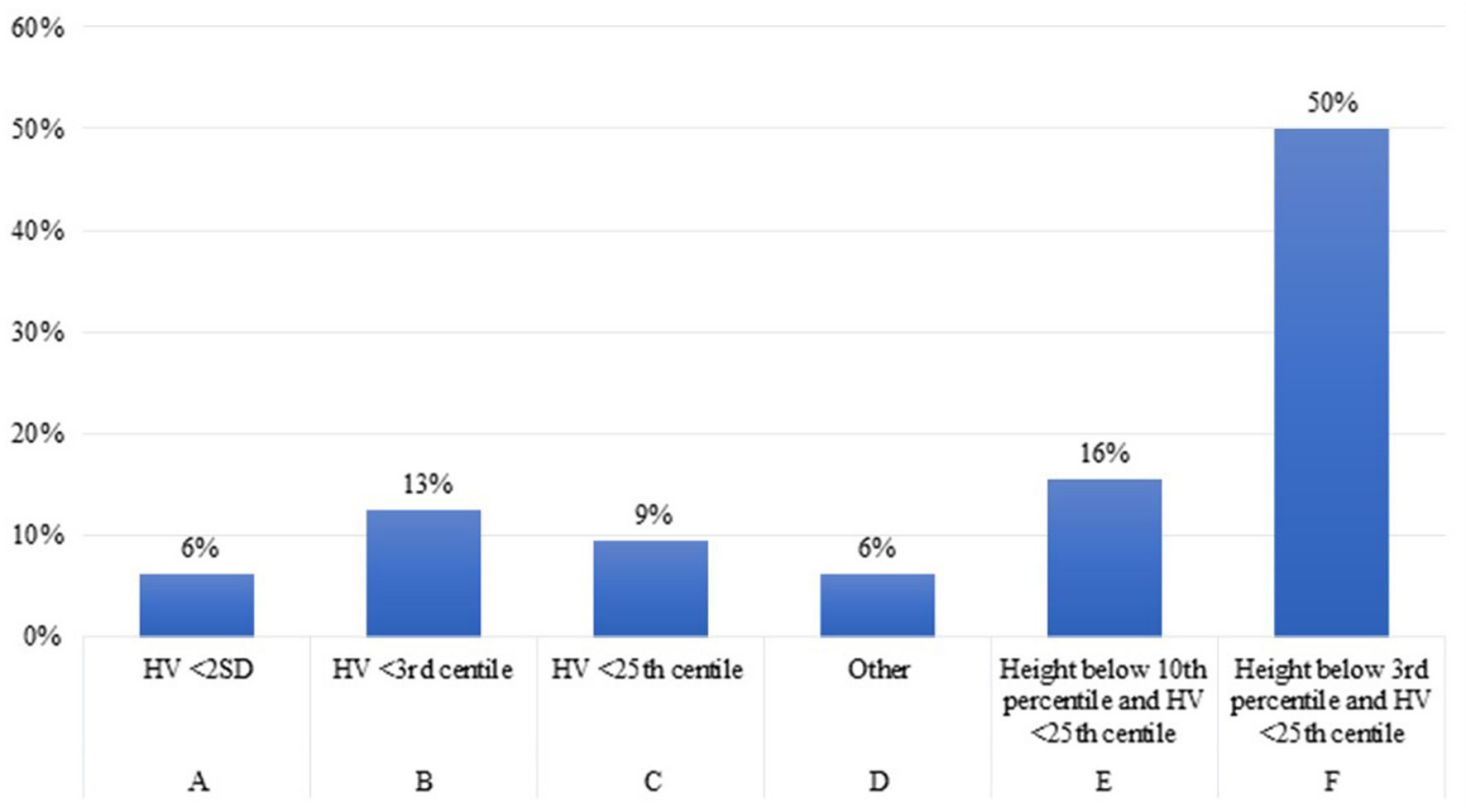
Parameters used to initiate rhGH treatment in patient over 2 years old with CKD.

Twenty one of the 32 experts (66%) reported to start rhGH therapy in children older than 2 years with CKD when height falls below the third percentile and height velocity (cm/year) is below the 25 percentile (Figure 3).

This is in line with the consensus statement of Drube et al. where the experts defined persistent growth failure as a height below the third percentile for age and sex and a height velocity below the 25 percentile, once other potentially treatable risk factors for growth failure have been adequately addressed (and provided the child has growth potential) (2). In the previous consensus (25), Mahan et al. defined growth failure as height velocity below 2 SD or height below the third percentile. It is reasonable to assume that 50% of the experts, in this case, followed the previous recommendations (25), while another 16% agreed with the more recent report by Drube et al. recommending to start rhGH treatment in children with height between the third and the 10th percentile (2).

**Question 4**. Below which eGFR level (ml/min/1.73 m^2^) do you start rhGH treatment in children older than 1 year with growth failure and CKD, once other factors related to growth failure have been addressed?

**Figure 4:**
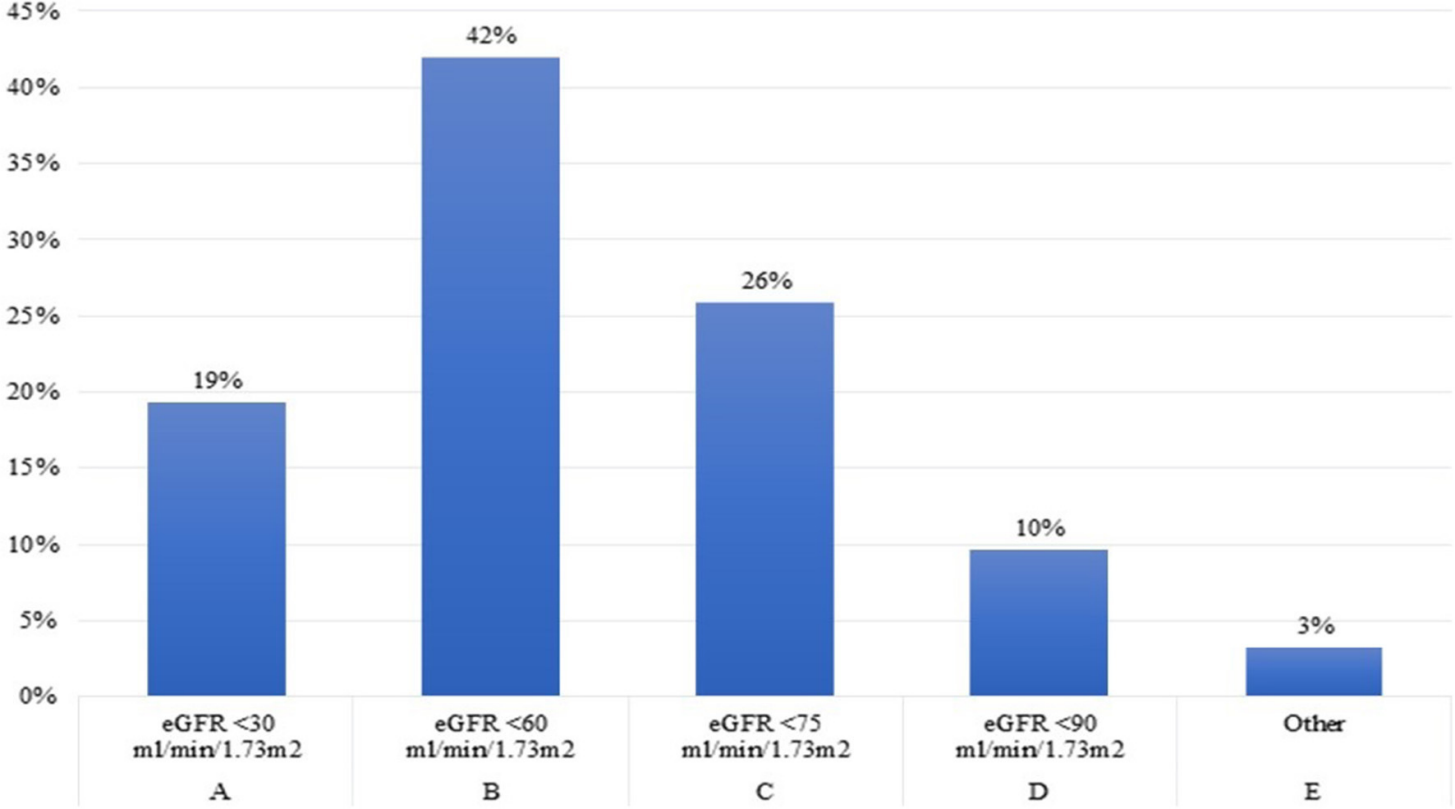
eGFR level at which rhGH treatment is started in children over 1 year old with different.

Forty-two per cent of the experts conveyed to start rhGH treatment at a eGFR level below 60 ml/min/1.73 m^2^, as reported by the recently published recommendations (2), while another 26% found appropriate to start treatment at a eGFR level below 75 ml/min/1.73 m^2^, as suggested by the 2006 Consensus (25). Thirty per cent of the pediatric Endocrinologists assumed that rhGH treatment should be started at an eGFR level of <30 ml/min/1.73 m^2^ (Figure 4). These results underline the importance of a closer cooperation and exchange of information within the multidisciplinary team involved in the care of growth failure of pediatric CKD patients.

**Question 5**. What is the parathyroid hormone (PTH) level in children with growth failure and CKD above which you believe that rhGH treatment should not be started?

**Figure 5:**
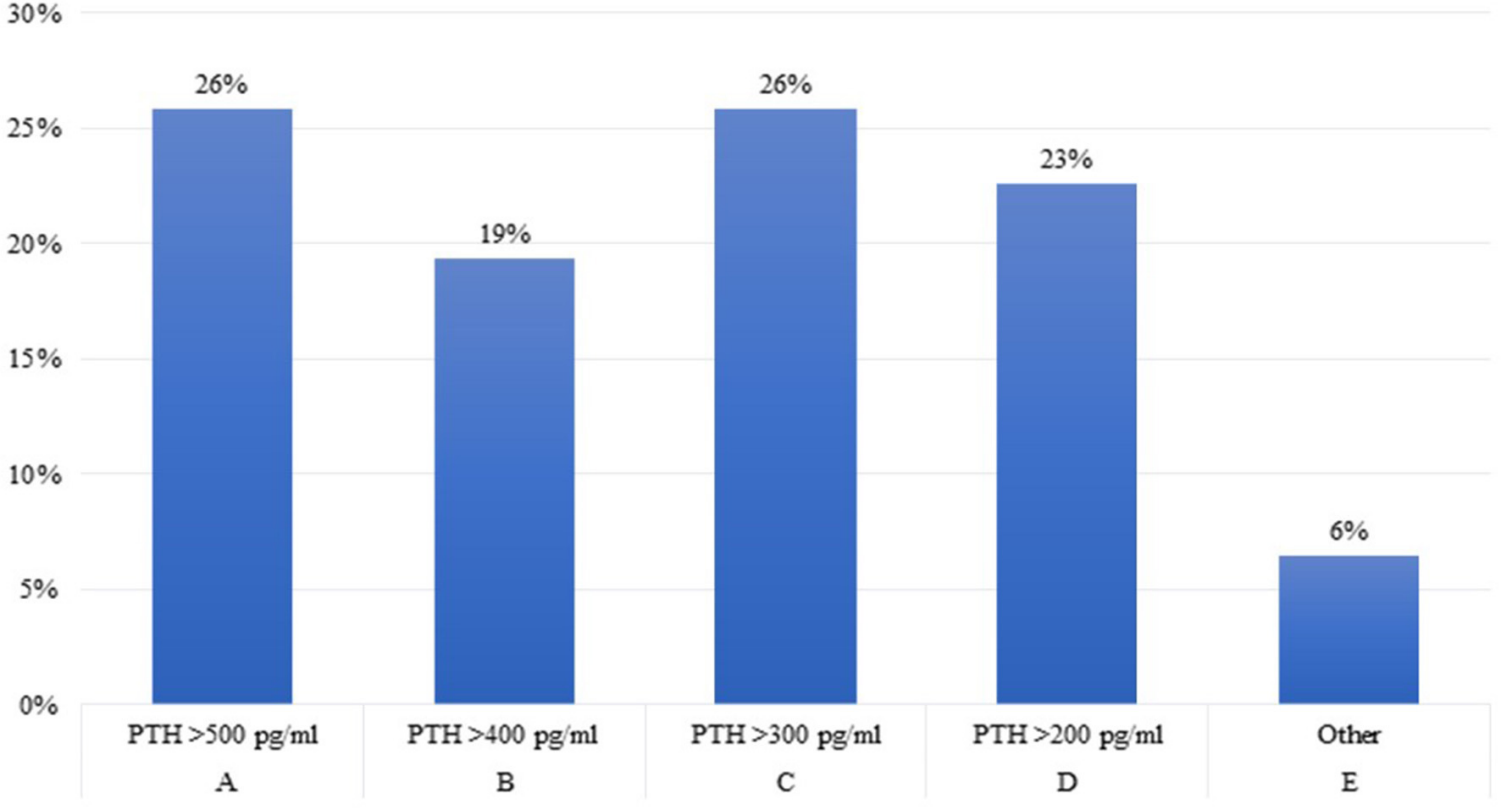
rhGH treatment and PTH.

A large disparity of opinions emerged between experts regarding serum PTH levels above which rhGH treatment should not be started in children with growth failure and CKD, with only 26% of them complying with the 2019 clinical practice recommendations (2) (Figure 5). In fact, the recommended PTH target values by CKD stage differ widely as evidenced by both the 2017 KDIGO guidelines (1), and the recent paper (2) of a pros and cons debate on this issue (28), suggesting to use trends rather than absolute target values to determine treatment decisions in children with CKD; In particular, patients with mean PTH levels >500 pg/ml showed a significant loss in height SDS compared with children with lower PTH levels (−0,28 vs. −0.05 SDS per year) (29).

Secondary hyperparathyroidism plays an important role in the development of the complex chronic kidney disease-associated mineral and bone disorders (CKD-MBD), with consequent metabolic bone diseases resulting in growth failure. In the treatment of other non-renal diseases, satisfactory growth can be achieved when PTH levels are within the normal range or slightly higher. Despite the relationship between longitudinal growth and serum PTH levels remains unclear, available data suggest that avoiding the development of a significant bone disease through an adequate control of PTH levels may improve linear growth by a small increase of height SDS (0,09 SD per year) (30).

Furthermore, children on peritoneal dialysis with mean PTH levels > 500 pg/ml showed a significant loss in height SDS (mean −0.28 SDS per year) (29). Finally, severe secondary hyperparathyroidism is associated with an increased risk of slipped capital femoral epiphysis in children with CKD (29). Therefore, it is essential to treat CKD-MBD before starting rhGH therapy, in agreement with current guidelines (1, 31). An increase in serum PTH levels has been reported in children receiving rhGH therapy as a consequence of both direct stimulation of PTH secretion by the parathyroid glands and slight changes in calcium and phosphate homeostasis (2). We believe that rhGH therapy should be discontinued in patients with persistent severe secondary hyperparathyroidism (PTH >500 pg/ml) and that it can be resumed when PTH levels return to the desired range (1, 32).

**Question 6**. Do you think corticosteroid therapy represents a limit to start/continue treatment with rhGH in children with growth failure and CKD?

Sixty-eight percent of the experts considered only high-dose corticosteroid therapy as a contraindication to start rhGH treatment in children with growth failure and CKD, compared to 26% who did not consider corticosteroids as a limit.

Long-term steroid therapy may affect growth by several mechanisms, such as preventing pulsatile GH secretion, inhibiting hepatic production of IGFI, and by peripherally interfering with cartilage metabolism, bone formation, nitrogen retention, and calcium metabolism. Poor growth outcomes observed in the post-transplantation population are associated with a high number of factors, including corticosteroid administration, decreased eGFR, and an abnormal GH–IGF-I axis (25). Drube et al. (2) recommended that rhGH therapy is considered for pediatric renal transplant recipients for whom expected catch- up growth cannot be achieved by steroid minimization or for patients in whom steroid withdrawal is not feasible owing to high immunological risk, particularly in children with suboptimal graft function (estimated eGFR <50 ml/min/1.73 m^2^).

**Question 7**. In which renal disease do you start rhGH treatment in CKD children with a growth deficit, once other factors related to reduced growth rate have been addressed, and independently of eGFR reduction?

**Figure 6:**
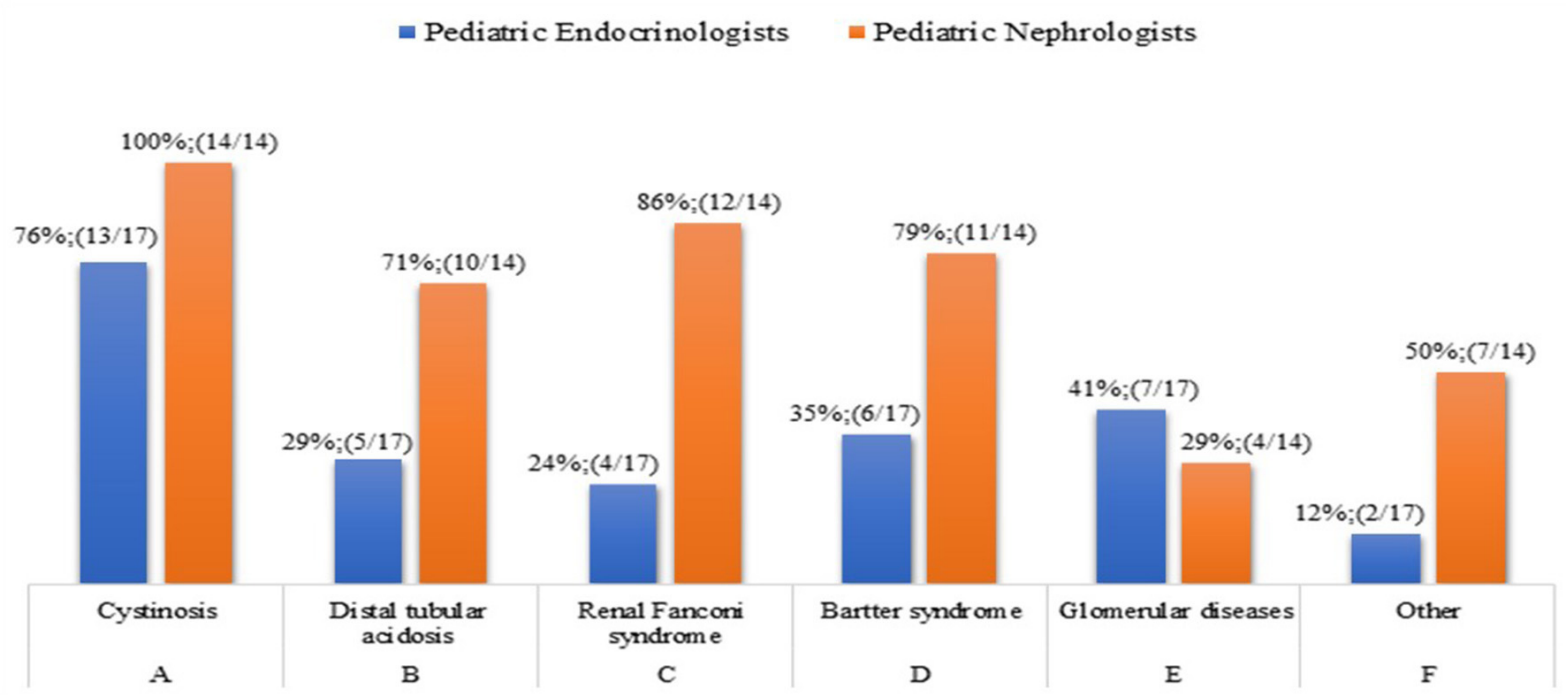
Diseases that leads to growth failure in nephropathic patients.

For patients with metabolic diseases there are recommendations on when to start rhGH treatment aimed at both catch-up growth and weight gain. The diseases associated with growth failure, independently of eGFR reduction, are reported in Figure 6. Because the question allowed a multiple-choice answers, pediatric Endocrinologists and Nephrologists agree that cystinosis leads to growth failure in nephropathic patients, and 27 out of 31 physicians are in favor of rhGH treatment. On the contrary, the results of the survey highlighted that Endocrinologists rarely start rhGH treatment in patients with diseases associated with growth failure, while an average of 72% of pediatric Nephrologists were in favor of rhGH treatment of distal tubular acidosis, renal Fanconi syndrome, and Bartter syndrome. rhGH therapy has a role in the treatment of growth failure in some patients with Bartter syndrome (33) and Drube et al. (2) recommended rhGH treatment in children with CKD due to nephropathic cystinosis who have persistent growth failure (height below the third percentile for age and gender and height velocity below the 25th percentile). rhGH in these patients should be considered at all stages of CKD.

The wide difference of opinion between Endocrinologists and Nephrologists may be due to the unclear indications in the 2006 Consensus Statement (25) in the assessment and treatment of short stature in pediatric patients with CKD, or because the most recent recommendations (2) have not been implemented into clinical practice yet.

**Question 8**. Would you start treatment with rhGH in a pubertal adolescent (Tanner III–IV) with CKD and growth failure, but with bone age at least 1 year less than the chronological age?

Eighty-seven percent of the experts would start treatment with rhGH in adolescent patients in advanced puberty phase with CKD and GHD, in which growth cartilage is not yet closed, but with bone age of at least 1 year less than their chronological age; 13% of the participants will never start rhGH at puberty. It is assumed that in late-pubertal adolescents, rhGH should be stopped if height velocity drops below 2 cm per year and/or epiphyseal growth plate closure is evident on hand radiography, as no further growth potential can be expected (2).

Few controversial data have been reported on the benefits of rhGH treatment in pubertal patients with CKD (18, 20, 34, 35). In one study, no effects on adult height were observed (20), while growth recovery was indeed reported in patients with CKD who were in the early stage or late puberty when treatment was started with rhGH (20). Height gain was reported by others (18, 34) and a height gain up to 1.0 SDS was also documented in a post-marketing database in patients with CKD who were either in the early or late puberty (35). None was a randomized trial suggesting the need for further information.

**Question 9**. Would you start treatment with rhGH in a child with growth failure who had received a kidney transplant since at least 12 months?

In children who have received a kidney transplant since at least 1 year, 78% of the experts (Table 2, A-B) agreed to start rhGH treatment when growth failure is confirmed (36), and half of them believed that rhGH treatment should be started even in transplanted patients with normal kidney function. However, Table 2 shows that there is a wide difference between the response of pediatric Nephrologists and Endocrinologists. The former were much more keen to treat with rhGH, while Endocrinologists were less willing to start treatment. There may be different reasons for this attitude, including either a low level of diffusion - among Endocrinologists - of the recent recommendations by the European Society for pediatric Nephrology working groups for rhGH treatment in CKD children (2), and the prescription limitations imposed by the Italian Medicines Agency (AIFA) note 39 (37). The recent recommendations (2) suggested growth monitoring for at least 1 year post-transplantation before rhGH therapy is considered in order to allow for spontaneous catch-up growth. rhGH therapy is considered for pediatric renal transplant recipients when the expected catch-up growth cannot be achieved by steroid minimization, or for patients in whom steroid withdrawal or tapering is not feasible owing to high immunological risk (2).

**Table 2:** Initiation of rhGH therapy in pediatric patients 1 year after kidney transplantation.

**Answers**	**Endocrinologists**	**Nephrologists**	**Total (%)**
A) Yes, even with normal kidney function	2	10	12 (39%)
B) Yes only in case of persistent CKD	10	2	12 (39%)
C) Never	0	2	2 (6%)
D) Other	5	0	5 (16%)
Total	17	14	31 (100%)

**Question 10**. Would you start rhGH treatment in a child with growth failure who has undergone kidney transplant since at least 12 months in the presence of chronic rejection or recurrence of underlying disease?

The group of experts generated two opposing views (42% in favor vs. 42% against starting rhGH). Pediatric nephrologists were clearly in favor of initiating rhGH therapy, while pediatric endocrinologists were mainly reluctant. However, there was an almost unanimous opinion on the need to consider the personalized treatment to be taken on the basis of a specific evaluation of each individual case. Factors such as steroid regimen, eGFR, patient age and pubertal stage, and the type of underlying disease that could influence the decision should be considered.

**Question 11**. When do you think it is necessary to discontinue rhGH treatment in patients with CKD treated for growth failure, in the absence of adverse effects due to rhGH therapy?

The discontinuation of rhGH treatment represents an area of controversy and may varies between pre-pubertal and post-pubertal/adolescents. Discontinuation of rhGH is recommended by some authors in patients who are poor responders over a period of at least 6 months (height velocity <2 cm/year), despite adherence to daily subcutaneous injection regimen (2, 33). According to others, therapy should be continued until the patient has reached genetic target height. In pediatric patients with CKD, and in pre-pubertal and pubertal renal transplants, improvement in height SDS attained with rhGH treatment was maintained even after its discontinuation (38).

**Figure 7:**
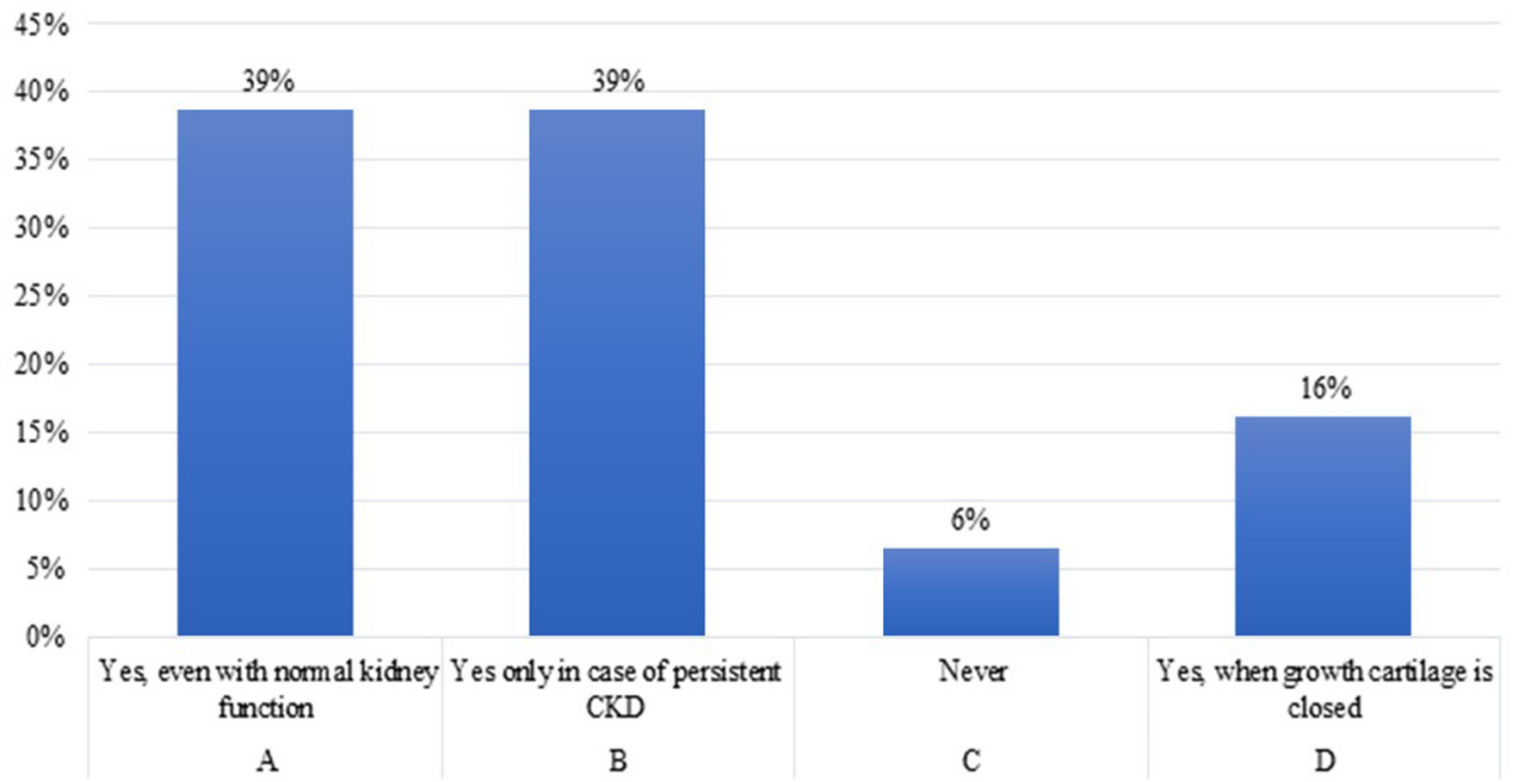
Discontinuation of rhGH administration in pediatric patients with CKD.

In the absence of adverse effects/complications due to rhGH therapy, 32% of experts were in favor of the statement by the recent recommendations (2) and agreed to discontinue rhGH treatment when the patient reaches his or her genetic target height; 52% of experts declared to discontinue treatment with rhGH when height velocity is below 2 cm/year as indicated by Drube et al. in late pubertal adolescents (Figure 7).

## Discussion

**Figure 8:**
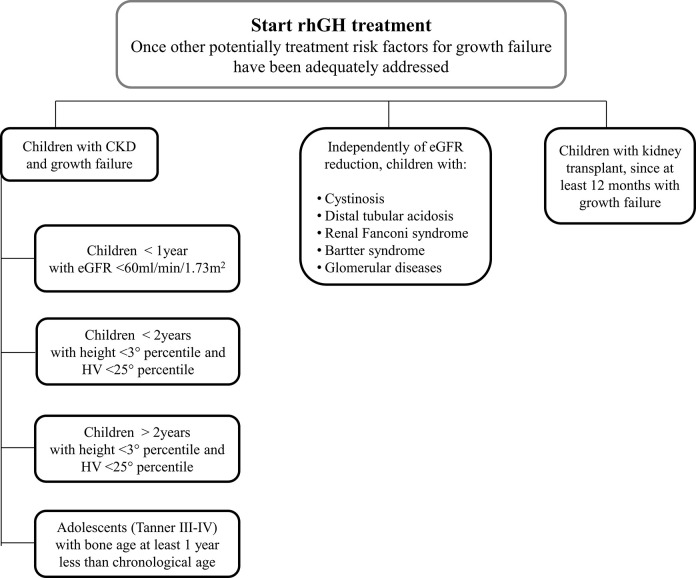
A proposed flowchart for the management of patients with chronic kidney disease and growth failure.

This survey is based on the current clinical practice of Italian pediatric Endocrinologists and pediatric Nephrologists who are involved in the care of CKD patients with growth failure. It provides important clues on the management of such patients and describes how the great majority of Italian experts behave in the clinical practice of CKD (Figure 8). In the early nineties, the multicenter, randomized, double-blind, placebo-controlled study of Fine et al. (36), showed that rhGH therapy could significantly improve the growth of children with CKD. Clinical studies (18, 23, 39–43) demonstrated the safety and efficacy of rhGH therapy in promoting height gain in children with CKD on dialysis or after kidney transplantation.

One of the first retrospective study on the use of rhGH in infants with CKD from the first year of life onwards reported that early hormonal treatment can accelerate increases in weight and height velocity, allowing kidney transplantation at an early age (43). The only adverse event observed was the tendency to develop hyperparathyroidism, which required treatment with higher doses of 1.25 (OH)_2_-vitamin D (44). During the first 12 months of therapy, children with CKD and growth failure, despite good metabolic and nutritional control, benefit from rhGH treatment with no adverse effects; the study by Santos et al. (25) showed that a minority of infants with CKD do not grow adequately despite proper nutrition and metabolic and clinical control, while a response to rhGH treatment with a significant improvement in body length has been observed.

In 2012 a Cochrane review of 16 studies with a total of 809 children enrolled showed that rhGH treatment (28 IU/m^2^/week) compared to placebo or no specific therapy resulted in a significant increase in 1-year height SDS as well as 6-month and 1-year growth velocity. The growth velocity, although declining over time, remained significantly greater than in untreated children during the second year of therapy. Thus, 1 year of 28 IU/m^2^/week of rhGH in children with chronic kidney disease determined a 3.88 cm increase in height velocity above that of untreated patients (45). There are pharmacoeconomic issues related to r-hGH treatment and the costs of competing r-hGH treatment options present different scenarios based on the different devices available including total costs, wastage costs, cost per mg and costs per cm gained with a mean of 4.700 Euro per cm gained (46).

The current use of rhGH therapy in CKD children remains rather infrequent and this could be at least partially due to the lack of clear guidelines for physicians concerning the initiation and follow up of rhGH therapy and the interpretation of height gain in treated patients. In a randomized controlled trial of the NAPRTCS (North American Pediatric Renal Transplant Cooperative Study), the efficacy of rhGH treatment in improving growth of pediatric patients undergoing renal transplantation was confirmed, without the occurrence of adverse events including acute rejection episodes during the first year of the study (40). A more recent work by Drube et al. recommends - in children who have received kidney transplant and fulfill the growth failure criteria - to start rhGH therapy 1 year after transplantation if spontaneous improvement of growth does not occur and steroid- free immunosuppression is not a viable option (2). As far as other conditions including Bartter syndrome, a group of rare autosomal recessive renal disease characterized by hypokalemic hypochloremic metabolic alkalosis, it has been shown that some patients may benefit from rhGH treatment suggesting that rhGH may have a role in the treatment of such condition. However, based on the Italian National Health Care System regulations for the use and reimbursement of rhGH (note 39 of the Italian Drug Agency, AIFA) (37), rhGH therapy is considered off label for the treatment of this group of diseases. In addition, the National regulations allow to treat “height deficit in patients with chronic renal failure” without defining the auxological parameters of short stature and the stage of chronic renal failure requiring rhGH therapy. The panel agreed that adverse effects of rhGH treatment are rare and may include glucose intolerance and increased cranial pressure, while the benefits of rhGH treatment and improvement of height (47) should be weighed against the burden of daily subcutaneous injections on an individual basis.

The expert group underlies the importance of multidisciplinary management of CKD children with growth failure and that there currently is no single, optimal approach and decision-making process for initiating rhGH therapy. Indeed, the low level of validation of the proposed algorithm for the assessment and treatment of growth failure in children with CKD in the 2006 (25) and recent consensus, as evidenced by the results of the survey, suggest that there are issues that have not been addressed and that the multidisciplinary approach remains essential for taking the best decision.

This multidisciplinary approach can also help overcome some poorly established prescription schemes in the treatment with rhGH of children with CKD and growth failure. Such prescription models are part of established clinical practices in real life in each category of specialists and maybe - at least in part- related to the slow acquisition of the recommendations of the most recent guidelines. What the experts agreed on is that successful therapy is based on early diagnosis and prompt intervention and that it is maintained through a strong multidisciplinary effort and continuous empowerment of the patient and the caregivers. Although current guidelines (1, 2) recommend rhGH therapy in all children with CKD and growth failure, its use varies widely worldwide, and rhGH is prescribed in a relatively small number of patients and rarely after renal transplant. The percentage of rhGH use 2 years after registration in the North American Pediatric Renal Trials and Collaborative Studies (NAPRTCS) registry was 22, 33, and 3% in children with chronic kidney disease, dialysis and transplantation, respectively, with a height SDS < -1 and age <17 years (42). The widespread underutilization of rhGH deserves further investigation.

In conclusion, the results of this survey confirmed that Italian pediatric Endocrinologists and pediatric Nephrologists follow guidelines in the management of children with CKD and growth failure, but these results bring to attention some issues that had not been considered by the 2006 and the most recent recommendations. A multidisciplinary approach and a mutual collaboration between specialists will allow to adapt the current recommendations, with the aim of improving patient care, and updating national rules for the prescription of rhGH according to patient's unmet needs. Finally, we believe these results are useful to develop national surveys in order to promote a broader international meeting that can help sharing expert perspectives and provide guidance and reference for the rational use of rhGH that are more practical from those proposed by the classical guidelines.

## Data Availability Statement

The raw data supporting the conclusions of this article will be made available by the authors, without undue reservation.

## Ethics Statement

Ethical review and approval was not required for the study on human participants in accordance with the local legislation and institutional requirements. Written informed consent for participation was not required for this study in accordance with the national legislation and the institutional requirements.

## Author Contributions

MC, MM, and EV participate to the design of the study, analyzed the results of the survey, drafted, and revised the manuscript. VC, LC, CE, CG, MG, LG, AK, GM, LM, MP, CP, and SP participate to the design of the survey, analyzed the results of the survey, and revised the manuscript. All authors approved the final manuscript as submitted and agree to be accountable for all aspects of the work.

## Conflict of Interest

The authors declare that the research was conducted in the absence of any commercial or financial relationships that could be construed as a potential conflict of interest.
